# A Brief History of Colour, the Environmental Impact of Synthetic Dyes and Removal by Using Laccases

**DOI:** 10.3390/molecules26133813

**Published:** 2021-06-22

**Authors:** Leidy D. Ardila-Leal, Raúl A. Poutou-Piñales, Aura M. Pedroza-Rodríguez, Balkys E. Quevedo-Hidalgo

**Affiliations:** 1 Grupo de Biotecnología Ambiental e Industrial (GBAI), Laboratorio de Biotecnología Molecular, Departamento de Microbiología, Facultad de Ciencias, Pontificia Universidad Javeriana (PUJ), Bogotá 110-23, DC, Colombia; ardilaleidy@javeriana.edu.co; 2 Grupo de Biotecnología Ambiental e Industrial (GBAI), Laboratorio de Microbiología Ambiental y de Suelos, Departamento de Microbiología, Facultad de Ciencias, Pontificia Universidad Javeriana (PUJ), Bogotá 110-23, DC, Colombia; apedroza@javeriana.edu.co; 3 Grupo de Biotecnología Ambiental e Industrial (GBAI), Laboratorio de Biotecnología Aplicada, Departamento de Microbiología, Facultad de Ciencias, Pontificia Universidad Javeriana (PUJ), Bogotá 110-23, DC, Colombia; bquevedo@javeriana.edu.co

**Keywords:** history of colour, synthetic colourants, natural colourants, environmental impact, laccases, biological treatment, coloured wastewater

## Abstract

The history of colour is fascinating from a social and artistic viewpoint because it shows the way; use; and importance acquired. The use of colours date back to the Stone Age (the first news of cave paintings); colour has contributed to the social and symbolic development of civilizations. Colour has been associated with hierarchy; power and leadership in some of them. The advent of synthetic dyes has revolutionized the colour industry; and due to their low cost; their use has spread to different industrial sectors. Although the percentage of coloured wastewater discharged by the textile; food; pharmaceutical; cosmetic; and paper industries; among other productive areas; are unknown; the toxic effect and ecological implications of this discharged into water bodies are harmful. This review briefly shows the social and artistic history surrounding the discovery and use of natural and synthetic dyes. We summarise the environmental impact caused by the discharge of untreated or poorly treated coloured wastewater to water bodies; which has led to physical; chemical and biological treatments to reduce the colour units so as important physicochemical parameters. We also focus on laccase utility (EC 1.10.3.2), for discolouration enzymatic treatment of coloured wastewater, before its discharge into water bodies. Laccases (p-diphenol: oxidoreductase dioxide) are multicopper oxidoreductase enzymes widely distributed in plants, insects, bacteria, and fungi. Fungal laccases have employed for wastewater colour removal due to their high redox potential. This review includes an analysis of the stability of laccases, the factors that influence production at high scales to achieve discolouration of high volumes of contaminated wastewater, the biotechnological impact of laccases, and the degradation routes that some dyes may follow when using the laccase for colour removal

## 1. Introduction

The textile, paper, and leather industries are responsible for discharging a large volume of coloured wastewater into water bodies [1]. Service providers, including hospitals, universities [2] and the food industry employs synthetic dyes [3]. Most dyes, neither maximum discharge limits nor toxicological effects on the environment and human health are known [4]. In general, country regulations set a colour limit but do not define specific limits for dyes and sometimes only require colour measurement and do not require coloured water limits [5,6].

Dyes cause harmful effects on the environment, even in low concentrations. In addition, there are other highly toxic compounds in the discharge of coloured wastewater that increase environmental problems [4]. However, it is known that dumping dyes into water bodies decreases the passage of sunlight [7], increases biochemical (BOD) and chemical (COD) oxygen demand, prevents photosynthesis and inhibits plant growth. Synthetic dyes are recalcitrant, bio-accumulative, toxic, mutagenic, and carcinogenic [8].

Treatments used for colour removal (chemical, physical, biological or hybrid); have been highly effective in removing colour [9]. However, chemical and physical treatments have the disadvantage of generating sludge that is difficult to handle, expensive and requires large treatment areas [1]. On the other hand, the anaerobic process is environmentally friendly, low cost and generates less sludge concerning aerobic treatments [10,11]. 

Microorganisms such as fungi, bacteria, yeasts and algae can discolour and even completely mineralize dyes [12,13]. White rot fungi are the most efficient at breaking down synthetic dyes because they produce enzymes that catalyze the dye removal and degradation reactions [14]. The colour removal efficiency is under the control of pH, nutrient load, treatment time, aeration, C/N ratio, biomass morphology, inoculum concentration, co-substrate addition, and the production of toxic by-products [2,15]. Due to some phenolic compounds inhibit fungal growth during bioremediation processes [16], the enzymes are an alternative to reduce the pollution and the environmental impact of industrial effluents they generate [17]. 

Enzymes have high catalytic efficiency, high or low substrate specificity (managed at convenience), require less reaction time. Concerning chemical processes, the low energy consumption, the reaction conditions are easy to promote and do not generate toxicity [11]. The catalytic potential of enzymes has allowed their use in the food and beverage, paper, cosmetic, pharmaceutical, detergent, textile, leather, wastewater treatment, and organic and polymer synthesis industries [18]. 

Laccases (EC 1.10.3.2) are enzymes with high catalytic efficiency [19,20] and have been applied in the delignification of lignocellulosic compounds, in bio-pulping and bio-bleaching, in the transformation of dyes, in the treatment of wastewater and the degradation of recalcitrant compounds, among others [11,20,21,22,23,24,25,26,27]. 

Laccases are multi-copper oxidase enzymes found in fungi, plants, insects and other natural sources [20]. During the enzymatic reaction, peroxidases generate hydrogen peroxide (H_2_O_2_) but laccases water (H_2_O) [28]. It is one of the reasons for laccase biotechnological interest. Laccases catalyze the oxidation of a wide variety of organic-aromatic compounds [20], is amplified by the addition of redox mediators [23,29,30]. The catalytic potential of enzymes is related to their structural stability (3D) [31]. 

At present, more than 100 laccases of Basidiomycetes and Ascomycetes have been purified and characterized. *Pleurotus* sp., can degrade synthetic dyes and other pollutants such as polycyclic aromatic hydrocarbons, pesticides, polyethylene, explosives and antibiotics [25,26,32,33,34,35,36]. *Pleurotus ostreatus* can produce among eight to eleven different laccase isoenzymes [20,37]; however, due to the complexity of liquid culture, growth time, production logistics and cost of solid culture, the larger-scale production using mushrooms is limited. 

In this order, the production of heterologous laccases using yeast reduce production costs and improves productivity [20,38,39]. The methylotrophic yeast *Pichia pastoris* constitute a successful expression system for the production of recombinant proteins [40,41] due to its high level of expression, the ability to use different carbon sources and the fact that it responds to various cultivation strategies for the production of the metabolites of interest [42,43,44,45,46]. 

Just as the action of laccases on different types of pollutants and several production conditions has studied, it is crucial to know the best storage conditions to favouring their stability and half-life [12,47,48]. Enzyme stability studies allow predicting when the enzyme activity loss has begun. It is crucial to consider that, even under optimal storage conditions, it is reasonable that structural and functional changes occur in the molecules [49].

Colours are frequent in our life, but sometimes we ignore the social-historical phenomenon that made colour an essential element of expression and communication for human beings. Colour has influenced people perception and has played a crucial role in social and natural environment acceptance. This review attempts to show, from a historical starting, the importance of natural dyes and how the industrial progress of colour increased their lead to synthesis and caused a negative environmental impact. This review shows the need to know about synthetic dyes and how their discharge into water bodies causes an imbalance in the ecosystem. In addition, we describe some strategies to eliminate synthetic dyes in wastewater and the strategies to mitigate the negative environmental effect, highlighting the application of laccases as a viable and economical alternative to treat coloured effluents before discharge into water bodies.

## 2. History of Dyes

Colour has been used since prehistoric times as a symbolic art of society, as a form of visual communication and as a mechanism of expression, associated with the cultural evolution of humanity [50,51,52], as the use of colour in different prehistoric environments has been linked to the artistic and cognitive development of individuals [50,53]. Some experts believe that colour was associated with a symbolic system spread and shared by many societies over different periods [54].

**Figure 1:**
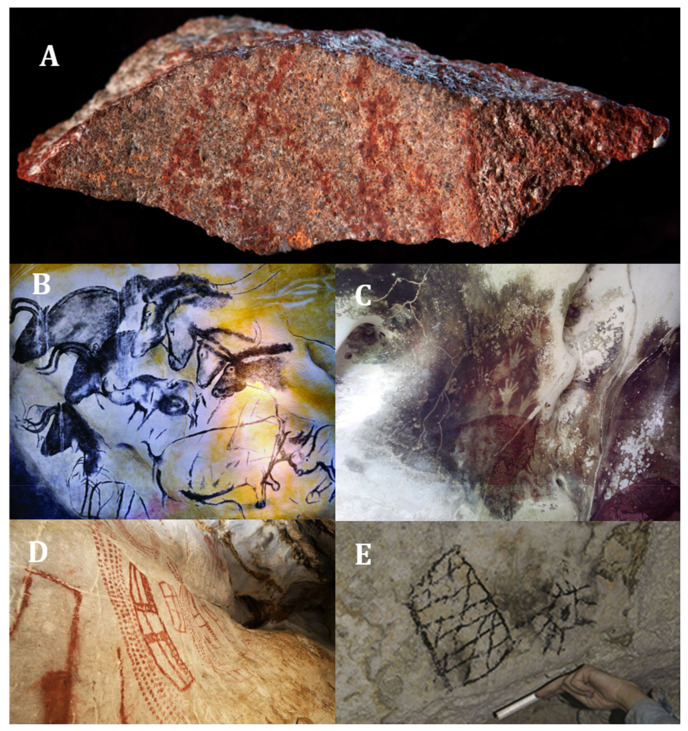
Pigments used in some caves with cave paintings made during the Middle Stone Age. (**A**) Ochre pigment in Bombs Cave, Southern Cape Town, South Africa (https://www.nationalgeographic.com/science/article/news-ancient-humans-art-hashtag-ochre-south-africa-archaeology Accessed on: 28 May 2021) [59]. (**B**) Coal pigment in Chauvet Cave, France (https://www.newyorker.com/magazine/2008/06/23/first-impressions Accessed on: 28 May 2021) [58]. (**C**) Pettakere Cave, Maros, Indonesia (https://reydekish.com/2015/09/21/cuevas-de-indonesia/ Accessed on: 28 May 2021). (**D**) Ochre pigment in El Castillo cave, Spain (https://www.efetur.com/noticia/expresiones-ser-human/ Accessed on: 28 May 2021), (https://www.donsmaps.com/castillo.html Accessed on: 28 May 2021). (**E**) Coal pigment in the Mona Island Cave, Puerto Rico [60].

Pigments arose from mixtures of soil or other materials with water, saliva or animal fat used to colour different surfaces [55]. The first pigments probably used to mark the skin [50] for ritual purposes or as insects or sun protection [56]. However, Duarte (2014) proposes that ochre could also be included in prehistoric humans’ diet, generating an iron supply essential for development [53]. On the other side, were found 57 red ochre parts in a 165,000-year-old cave [57] (Figure 1A). However, clear evidence for the use of pigments has observed in cave paintings on exposed rocks or in caves [52,55].

In the prehistoric record, the frequency of use of different pigments increased over time, although this occurred differently at several times and regions. The initial pigments use has traced back to the Middle Stone Age/Middle Paleolithic in Africa and Europe (150,000–30,000 BC) [54]. The use of pigments from ochre clay dates back 100,000 to 70,000 years, as be seen in Blombos cave in South Africa with parallel line engravings [58,59]. Other reports, dating back 40,000 years in the caves of Indonesia (Pettakere Cave, Figure 1C), Australia (Arnhem Plateau), France (Chauvet Cave, Figure 1B), Spain (El Castillo Cave, Figure 1D) and Romania (Caliboaia Cave) [58]. However, the use of these pigments in cave art extended to more recent times, as the last paintings found was made in the 15th century in different caves and caverns on the island of Mona (western Puerto Rico, Figure 1E) [60]. 

The extensive prehistoric record of pigments use indicates that red and black pigments derived from natural rocks or other geological components were present in almost all settlements and quarries from the Palaeolithic to the Upper Palaeolithic (35,000–10,000 BC) [54,61]. According to reports, iron oxide or ochre were the components for the colours red, orange and yellow and carbon for the colour black [62]. After this period, there was a rapid diversification in the use of ochre on different environments and objects, caused by the widespread ochre as a symbolic element [54]. 

The first stages of human civilisation began during the late Neolithic period (6000–3500 BC) and the Bronze Age (3000–1200 BC), due to prehistoric-man life changes, who changed from nomadic to sedentary, as a result of the development of agriculture [63]. With the development of civilisation, the pigments began to use in paints made on temple walls, tombs, ceramics or homes. They were used to dye textile fibres, although their use on skin and hair precise [64,65]. Pigments also highlighted the cuneiform tablet writing relief, developed during the civilisations rise in Mesopotamia (3000–2000 BC) [66]. However, depending on the purpose, the pigment produced had a different composition. For example, in the Mayan civilisation (2000 BC to 900 AD), pigments were used to decorate ceramics with red line motifs was enriched with iron and chrome, while the red pigment used for red and black decorations contain cerium [65]. 

In prehistoric times, access to colours other than ochre and black was difficult [67]. For example, the colour blue (highly valued and as expensive as gold) was initially only obtained from lapis lazuli deposits in Afghanistan [68], which led to different civilizations developing processes for obtaining the colours. The ancient Egyptians associated the colour blue with the sky and water; being the first to artificially produce a blue dye, known as Egyptian blue, developed between 2900–2750 BC during the fourth dynasty (2630 BC, Sneferu (Pharaoh) to 2500 BC, Shepseskaf (Pharaoh)), [64]. Egyptian civilizations used Egyptian blue frequently over the next few millennia. The golden age of the Egyptian blue use was probably the New Empire (1580–1085 BC) period, which coincided with the most productive artistic period in ancient Egypt [67]. 

However, the Egyptians were not the only ones to generate techniques for obtaining the colour blue. The Chinese developed the Han Blue pigment during the period of the Chinese War around 500 BC [69]; the Mayan civilization developed the Maya Blue pigment, which has reports of use for coloured structures of the Upper Preclassical period (350–150 BC) [70,71]. 

The purple colour also impacted different civilizations, and it was so exclusive that only people with wealth or power could wear garments dyed with that colour. The Phoenicians were the ones who stood out in the processing and commercialization of the purple colour “Tyrian”, which obtained from snails (*Murex brandaris* or *Murex trunculus*), and it believed that the development of this colour occurred at the end of the Bronze Age (1550–1200 BC) [72,73].

Obtaining colours from pigments or dyes was constant activity in the different civilizations, getting to obtaining different colours from minerals, vegetables or insects [55]. The Medieval Age (Middle Ages—5th to 15th centuries) was distinguished by the bright, clear, and well-defined use of colours [55]. During that age, the development of new dyes was scarce, in contrast with advances in the dyeing process and the transport of colourants across Europe [64]. In the Renaissance (15th to 16th centuries AD), the development of new dyes was similar to that of the Medieval Age. During these years, cultural and artistic progress encouraged the low-cost obtaining of widely used colours such as gold or gilding; developing gold substitute pigments since the Medieval Age. The gold colour depends on a thin sheet of metal that adhered to the surface [55].

In 1630, Cornelius Drebbel, mixed cochineal red (obtained from insects) with tin for improving the stability of natural dyes and producing the first dye, erroneously classified as synthetic [74]. In 1704, Diesbach, synthesized the first pigment, known as Red Lake and generates knowledge for some other synthesis. In 1788, Carl Scheele developed the Emerald Green or Scheele Green pigment, a dye with high toxicity since it was composed of copper aceto-arsenite, but it only used until 1960. In 1788, Carl Scheele developed the Emerald Green or Scheele Green pigment, a dye with high toxicity since it was composed of copper aceto-arsenite, but it only used until 1960 [55]. 

In 1826, Otto Unverdorben was the first to prepare aniline from the destructive distillation of indigo. Later research into new dyes included methodological processes with aniline without knowing it; however. August Wilhelm Von Hofmann described that all the substances studied were the same [75]. Henry Perkin developed and patented the first synthetic dye called Mauveine, derived from coal tar, in 1854 [64,76]. Perkin solved the manufacturing problem, marking the beginning of different procedures to produce new synthetic dyes [75]. 

The use of ingredients distilled from coal tar enabled the development of new dyes, and by 1869 some natural dyes such as Alizarin was replaced by synthetic dyes at a low price [64]. Since then, dye production has diversified, with reports of more than 100,000 synthetic ones [77]. At the beginning of the 20th century, dye production concentrated in Europe. Today, the largest producers and suppliers of fabrics worldwide are China and India [78].

## 3. Dyes and Classification

Dyes are natural or artificial substances that provide colour to different fibres used in the textile, pharmaceutical, food, cosmetic, plastic, photographic and paper and other industries [79]. Colourants can be pigments or dyes. Pigments are practically insoluble, and the particles that make them up range from 1–2 μm. Dyes are easily dissolved in water and have a particle size ranging from 0.025–1.0 μm [80,81]. The industrial advantages for the use of artificial dyes based on (i) being chemically stable over time, (ii) being inert to physical, chemical and biological degradation, (iii) being able to give colour to the fibre to be dyed through reproducible processes, maintaining the colour intensity [82] and (iv) are low cost [77]. 

Dyes absorb light in the visible spectrum (400–700 nm), have extended conjugation and one or more chromophores [83]. Chromophores contain heteroatoms such as N, O, and S and include bonds such as -N=N- (azo), =C=O (carbonyl), NO or N-OH (nitrous), -NO_2_ or NO-OH (nitro) and C=S (sulfur), [84]. Chromophore groups are unsaturated and consist of atoms or groups of atoms, in which the arrangement of successive single and double bonds resonate, thus allowing the absorption of light rays [85]. Synthetic dyes present a considerable structural diversity and therefore have very different chemical and physical properties [84]. Table 1 shows the main chromophores that influence the classification of dyes. In addition to chromophores, most dyes contain auxochrome groups, which are not responsible for colour but are for intensity (tone) and affinity for the fibre. Some of them are -NH_3_ (amine), -COOH (carboxyl), HSO_3_ (sulphonate) and -OH (hydroxyl) [84,86]. 

## 4. Industries and Sectors Applying Synthetic Dyes 

The dyes most frequently used at industrial scale are azo, anthraquinone, indigo, xanthene and triarylmethane [84]. However, the use of aromatic compounds with complex structures such as anthraquinones has been remarkable [1]. Most synthetic dyes are used in the textile and tanning industries to dye a wide variety of products. Besides, other industries, such as the cosmetics industry, the paper industry, the food industry, the pharmaceutical industry, and service providers, use synthetic dyes (Hospitals, Universities, among others) [78].

Wastewater type composition varies according to the discharge procedure and the origin of the dye into domestic wastewater (DWW) and non-domestic wastewater (nDWW), which includes wastewater from industrial, commercial or service activities. DWW may be incidentally contaminated, so the level of contamination may be low, but nDWW must be effectively stored and treated before final disposal [2].

The service provider sector uses dyes in biological staining techniques (hospitals—universities) [78]. Colourants found in wastewaters from service providers varies depending on the staining methodology carried out. For example, in biomedical research laboratories performing clinical protocols for staining biologicals fractions, such as Haematoxylin, Eosin Y, Rose Bengal or Auramine O are discarded. Although the volume of dye solutions is relatively small, the concentration is very high (between ~1 to 10 g L^−1^), which generates wastewater with high toxicity [95]. Other service providers often generate considerable volumes of wastewater that can be differentiated depending on the discharge procedure and the dye origin, as domestic wastewater (DWW) and no-domestic wastewater (nDWW). The DWW can be contaminated fortuitously, so the contamination level can be low, while nDWW are from industrial, commercial, or service activities. This wastewater should be stored in special containers that allow its collection for later treatment by specialized companies and carry out neutralization and final disposal [2]. 

The dyes used in the textile industry are those with the worst impact worldwide. The azo dyes have low cost, high intensity and colour fastness [4], which has led to them being the most frequently used class of dyes (~60%). The second dyes most used are anthraquinones, characterized by their dyeing performance, easy accessibility and low cost [96]. The dye selection depends on their affinity and bond stability with the fibre, diffusion, reactivity, cost and fixing characteristics [4]. In the paper industry, a large volume of wastewater came from handcrafted paper dyeing [97]. 

In the cosmetics industry, products such as lipsticks, blushers, eye shadows, eyeliners and nail polish contain one or more colourants (dyes or pigments) in a concentration of between 1% and 25 % to provide the desired colours [98]. However, the industry most important activity is the production of hair dyes, which accounts for about 80% of cosmetics in Europe [78]. 

Dyes in the food industry increase attractiveness or compensate for colour variations after food processing [78]. Dyes confer colour to carbonated drinks, fruit drinks, energy drinks, candies, cereals, desserts, snacks and others [99]. Currently, there are more than 60 known synthetic dyes for use in food; among the most common are the anionic dyes Sunset Yellow (E-110), Tartrazine (E-102) and Ponceau 4R (E-124) of the azo class; Solid Green FCF (E-143) of the triphenylmethane class, and Quinoline Yellow (E-104) of the quinethazones [100]. Although the type and concentration of dye allowed in food are under regulation in each country, different studies have shown the use of inadequate dyes at a concentration that exceeds the maximum allowed; this creates a risk for consumers [101,102,103]. 

Generally, food approved colours based on the Codex Alimentarius Commission (CAC) standards can also be used in the pharmaceutical industry [104,105]. Therefore, it is common to find dyes in pharmaceutical products such as Tartrazine (E-102), Sunset Yellow (E-110), Ponceau 4R (E-124), Azorubine/Carmoisine (E-122), Amaranth (E-123), Bright Blue (E-133) and Allura Red (E-129) [105]. Table 2 lists different industrial sectors, with the type of dye, its characteristics and some applications.

## 5. Impact of Synthetic Dyes on the Environment

The largest generator of coloured wastewater is estimated to be the textile industry [11,114]. Approximately 20% of the dye used for dyeing textile fibres is not fixed and is disposed of in the wastewater [115], resulting in a high level of pollution [115]. However, the environmental damage does not depend uniquely on the amount of dye discharged; but also depends on the dyes mixture with the other substances, all of them with toxic properties that make up the effluent from the industries [78,116]. 

Coloured wastewater sometimes containing dyes, visible to the naked eye (<1 ppm) [117,118], discharged into surface or groundwater bodies, cause a decrease in the concentration of dissolved oxygen in the water [14], increase the values of physicochemical and biological parameters such as the chemical oxygen demand (COD) [119], biochemical oxygen demand (BOD), total dissolved solids (TDS), total nitrogen (TN), total phosphorus (TFP), and non-biodegradable organic compounds. On the other hand, wastewater have a very fluctuating pH and heavy metals such as chromium (Cr), arsenic (Ar) and zinc (Zn) [85]. 

In general, synthetic dyes are not biodegradable due to their chemical properties and structure, generating an adverse effect on the environment [120]; most synthetic dyes are recalcitrant, carcinogenic and toxic for ecosystems [22]. On the other hand, the negative impact of dyes can be biomagnified, generating high contamination rates at high trophic levels [8]. However, the toxicity of each dye, must be assessed individually, as the damage they cause depends on the structure and exposure concentration [121], which means that dyes can persist for a long time (~50 years or more) in the environment [84]. The dyes persistence is closely related to their chemical reactivity, so unsaturated compounds are less persistent than saturated ones. The persistence of aromatic compounds increases as the number of chemical and halogen substitutions increases; the same happens for the persistence of dyes [85], demonstrating the relevance of assessing the degradation of dyestuffs individually and in combination. The most representative dyes in use belong to the azo, anthraquinone or triarylmethane classes [85,122], so this section of the review will focus on these three chemical dyes groups.

The azo class dyes have widely studied to knows their use and negatives effects. Between 60% to 70% of the azo dyes are toxic, carcinogenic and resistant to conventional Physico-chemical treatments [85]. The toxicity of azo dyes follows their chemical reduction and the subsequent formation of aromatic amines, such as benzidine, dimethoxy-benzidine and dimethyl-benzidine. The aromatic amines toxicity is due to their metabolic oxidation because the oxidation generates electrophilic reductive intermediaries (diazonium salts) that enable covalently bind to DNA. These compounds are mutagenic and cause diseases such as cancer. A variation of this mechanism is the chemical reduction of some of the azo bond (found in certain dyes) to the corresponding toxic aromatic mono-azo amine [7,96,123]. 

When azo ionic dyes discarded in surface or wastewater, they can bind to suspended organic matter by electrostatic interactions adhere to sediments or wastewater sludge, increasing the persistence [124]. Additionally, coloured water or contaminated sludge gets into contact with aquatic animals, transferring the toxic compounds through the food chain to humans, causing health disorders such as hypertension, cramps, nausea, bleeding, ulceration of the skin or the membranes and mucous membranes. Depending on exposure doses of dyes, may occur crucial damages to the kidney, reproductive system, liver, brain and central nervous system (CNS) [7,123]. Parrot et al., (2016) evaluated the effects of azo dyes on big-headed fish (*Pimephales promelas*) in the embryonic (larval) stage by comparing the dye effects at different concentrations. Authors found that the use of 25.4 mg L^−1^ and 16.7 mg L^−1^ of the azo dyes Disperse Yellow 7 and Sudan Red G, respectively, decreased the larvae survival, dying between four and ten days after hatch [125].

Anthraquinone dyes are considered the most toxic in spite they have widely used. Studies on biotransformation and toxicity of anthraquinones have been limited compared to azo dyes. Novotný et al., (2006) evaluated the toxicity of four dyes; two azo (Reactive Orange 16 (RO16); Congo Red (CR)) and two anthraquinones (Remazol Brilliant Blue R (RBBR); Disperse Blue 3 (DB3)), for which they used three species as a biological model, *Vibrio fischeri* (bacteria), *Selenastrum capricornutum* (microalgae), and *Tetrahymena pyriformis* (ciliate). Mutagenicity of dyes was determined using the Ames test by using Salmonella Typhimurium (His−). Authors found that the dye DB3 was the most toxic for all species and showed “in vitro” mutagenic effects in *S*. Typhimurium [126]. Some anthraquinoids dyes are very resistant to chemical oxidation due to their stability due to the resonance of their aromatic structure; this increases the permanence in the wastewater and results in textile wastewater with high percentages of anthraquinoids dyes [127]. 

Toxicological studies of other anthraquinones have generated different results. Blue Reagent 4 was considered phytotoxic, cytotoxic and genotoxic [128]. However, Acid Blue 80 (AB80) and Acid Blue 129 (AB129) evaluated at high concentrations did not generate a negative effect on larvae of big-headed fish (*Pimephales promelas*) after 14 days of exposure [125].

It is well known that triarylmethane class dyes severely affect metabolism, accumulate and penetrate the skin, are irritating if ingested or inhaled, induce carcinogenic effects, produce sarcomas and cause methaemoglobinaemia when species are overexposed [129]. An investigation carried out in water bodies in Belgium, to identify the level and accumulation of toxic dyes in the endangered wild species *Anguilla anguilla*, found that the eel was contaminated with dyes in 77% of the organs sampled and that between 25% and 58% of the samples contained the dyes Malachite Green (MG), Violet Crystal (CV) and Bright Green (BG) triarylmethane. Among the three dyes, MG has been the most studied because it is considered a multiorgan toxic, which affects the immune and reproductive system and has genotoxic and carcinogenic properties; effects that threaten this and other species in critical danger of extinction [130].

Chia and Musa (2014) used textile industry effluent (containing the dye Indigo) to assess the effect on growth, biomass production and phenotypic plasticity of the microalgae *Scenedesmus quadricauda*. The authors also demonstrated the environmental stress caused by the dye, as the growth rate of the microalgae decreased as dye concentration in the effluent increased and that at high dye concentration, the chlorophyll, cell density and dry weight of the microalgae were negatively affected [131]. 

However, the type of dye is not the unique one responsible for the harmful environmental effect. Croce et al., (2017) performed toxicity bioassays facing two species, *Daphnia magna* and *Raphidocelis subcapitata*, with 42 commercial dyes. They found that nine of the dyes were toxic to *D. magna* at concentrations below 100 mg L^−1^; in contrast, thirty of them were toxic for *R. subcapitata*, demonstrating the importance of sensitivity of some species and the different effects among dyes [132], (Table 3).

## 6. Treatment of Coloured Wastewater

The decrease of the colour units is one of the indicators for the treatment of wastewater containing dyes [140]; the most critical stage for effective treatment is the characterization of the effluent since it generates in the different industries, differ in terms of the discharge parameters established by countries [141]. Water discharge parameters indicate the quality and changes in wastewater when treatment is carried out [142].

Reducing the cost of treatment and removing the highest percentage of pollutants before discharged the wastewater into water bodies has been the focus of several studies, including chemical, physical, biochemical, biological and hybrid processes [9,140,143]. In this sense, treatments that combine physical, chemical and biological processes are usual to reducing pollutants in industrial effluents [140,141,144].

Among the three treatments types (biological, chemical, and physical), the elimination of dyes through physical processes is the most frequent. Physicals are simple and effective methods and involves adsorption, flotation, sedimentation, irradiation, membrane filtration (nanofiltration-ultrafiltration), and reverse osmosis (Table 4), [145]. These are non-destructive processes; however, depending on the treatment, difficult to remove and treat hazardous sludge can be generated. However, it is a viable option for dyes that generates more toxic products during their treatment than the original dye [143].

Chemical water treatments are expensive compared to physical or biological. Although some chemical treatments can be efficient in colour removal (Table 5) [145], the consumption of strong-oxidising substances, such as chlorine and ozone, for promoting colourant chemical degradation is so high. Thus, a scale-up process would generate a new problem, the need for specialised equipment with high cost and energy consumption [4].

The use of hybrid systems integrating physical and chemical treatments is frequent for colour removal as a strategy to reduce contamination. Photocatalysts combined with membrane systems allowed Methylene Blue (MB) degradation, with 83.3% removal in 370 min; achieved by controlling pH and salt concentration [149]. Photocatalytic degradation systems under visible light have employed to evaluate the degradation of Reactive Violet 5 (RV5), and 50Fe-TiO_2_ level achieved higher degradation efficiency (47.6%) in 9 h [155]. 

Biological methods are viable alternatives for the treatment of coloured wastewater because they are ecological and cost-effective. Depending on the treatment, they produce less sludge, which should not be used if the sludge adsorbs dyestuffs. In addition biological methods often generates non-hazardous metabolites or reach full-mineralization and consume low water [140]. However, some studies have found that biological methods require a long time, and some methods are ineffective in removing highly structured polymeric dyes with low biodegradability. Biological ones are not effective for some of the coloured wastewater due to the toxicity of commercial dyes for the organisms used in the process [151]. However, it has led to the evaluation of treatments that do not require the direct use of the microorganism, such as adsorption by dead microbial biomass (biosorption), living immobilized biomass and the use of free or immobilized enzymes [8,27,156].

Biosorption uses living or dead cells, these latter having advantages as bio-sorbents since they do not require nutrients, can be stored and used for long periods [157]. However, the biosorption treatment does not remove all dye concentration because it is trapped in the adsorption matrix; the reason for which subsequent treatment is necessary before managing the contaminated biomass discharge. It is a limitation for the treatment of large volumes of effluent [14]. Even so, biosorption has been widely used as a colour removal strategy, as the operating cost is low and hybrid systems allow to increase colour removal efficiency [8].

Almeida and Corso (2014) evaluated the biosorption of Procion Red MX-5B using *Aspergillus niger* biomass. They found that after three hours of incubation at 30 °C the discolouration was only 30%, and UV-Vis analysis showed no changes in the dye structure. In this study, biodegradation used the fungus *Aspergillus terreus*, which achieved 98% discolouration after 336 h of treatment [158]. Zuorro et al., (2017) has evaluated dead cells of the microalgae *Nannochloropsis oceanica*, finding that 72 h of contact with the RV5 dye allows reaching equilibrium, with a maximum adsorption capacity ranging from 75.9 to 115 mg g^−1^ [159]. Although the discolouration was efficient, the treatment time was not appropriate for application on a larger scale. Furthermore, treatment time and removal efficiency depend on factors such as, the organic load/colour ratio, temperature, oxygen concentration in the system, agitation, pH, salt and biomass concentration [9,96,159]. 

The type of microorganism for dye removal is crucial, as some bacteria, fungi or algae use some of the different enzymes they produce [160,161]. In general, enzyme-mediated biodegradation of dyes is efficient [15], as the catalytic activity increases the reaction speed, and little concentration of the enzyme is required. Therefore, microorganisms producing the proper enzymes can act on the dyestuffs and efficiently reduce or remove water pollution [123]. However, microorganisms can be easily affected by pH, temperature, chemicals, high salinity and toxic organic compounds [157]. The use of enzymes (without microorganisms) as an alternative treatment strategy for dyestuffs and other industrial pollutants is advantageous if it succeeds in adjusting the treatment conditions.

Enzymes are catalysts with varied specificity for the substrate; in general, their catalytic reactions produced lower toxicity by-products with high efficiency. Some of them (oxidoreductases) have the potential for different dyes treatment [1,123]. However, some authors have argued for reasons to limit the use of enzymes, as they have considered that their production is expensive [123]. However, advantages such as: being relatively easier to use, substrate specificity and easy regulation of catalytic activity and implementation remain attractive and promising [123].

Some of the enzymes used for dye degradation are lignin peroxidase (LiP, EC 1.11.1.14), manganese peroxidase (MnP, EC 1.11.1.13), azoreductase (EC 1.7.1.6) or laccase (Lac, EC 1.10.3.2), [160,161]. Each of these enzymes has its characteristics that define its biodegradation efficiency and working conditions. However, among the enzymes mentioned, laccases are characterized by not requiring hydrogen peroxide as occurs with LiP and MnP, nor do they require co-factors such as NADH_2_, NADPH_2_ and FADH_2_ for catalysis activation as occurs with azoreductases [160].

## 7. Laccases

Laccases (p-diphenol: oxidoreductase dioxide) are multicopper oxidoreductase enzymes [162] and catalyze the reduction of molecular oxygen (O_2_) to water (H_2_O) [163]. Laccase was first reported by Yoshida in 1883 [164], as part of the sap of the Japanese tree *Rhus vernicifera*. Laccases are ubiquitous oxidases present in plants, lignin-degrading fungi (Basidiomycetes, Ascomycetes and Deuteromycetes), [165,166,167], bacteria [168], algae [169], and insects [170].

Laccases are well-studied enzymes, and databases such as the National Center for Biotechnology Information (NCBI) report 191,500 items, where ~87% correspond to bacteria. However, biotechnological applications are focus on fungal laccases, probably to their high redox potential. Fungal laccases are involved in lignin degradation and mineralization, pigment synthesis, fruiting body formation, fungal morphogenesis, detoxification, sporulation and some pathogenesis processes [20,171,172]. In plants, laccases participate in the formation of insoluble protective barriers (laccases secretion), contributing to the defence of the plant, the polymerization of lignin [173], cytokinin homeostasis, polymerization of flavonoids in seed coatings and resistance to phenolic contaminants [169]. In bacteria, laccases are involved in melanin production, spore resistance, morphogenesis, copper detoxification and manganese oxidation [174,175]. In insects, laccases have the function of sclerosing and pigmenting the cuticle [170]. Finally, in algae, laccases are linked to the detoxification of phenolic compounds, the synthesis of biopolymers associated with the cell wall and the acquisition of nutrients through the transformation of lignocellulosic substrates [169].

## 8. Structure of the Active Centre of Laccases

The active site of laccase has four Cu^+2^, distributed in three sites [176], identified as Type I (CuT1), Type II (CuT2) and Type III (CuT3) copper, where CuT2 coppers and a CuT3 copper pair forms the trinuclear copper centre (TNC), [20,177,178]. Commonly, laccases have the same structural architecture, consisting of three cupredoxin-type domains, arranged sequentially (Domains 1, 2 and 3), where CuT1 is in Domain 1 and the copper that makes up TNC (CuT2 and two CuT3) join Domains 1 and 3 [20,177,179]. 

The CuT1 site is mononuclear and gives the enzyme its blue colour due to its spectroscopic characteristics. CuT1 is in Domain 3, approximately 6.5 Å below the surface of the enzyme; in a surface depression, delimited by one Domain 1 β turn and two Domain 3 β turns, involved in the binding of the substrate [180]. The coordination of CuT1 is different according to the microorganism origin.

Although several theoretical studies have shown that Cu (II) ions prefer square to flat and square to pyramid coordination [181], in most fungal laccases, the absence of an axial quarter ligand in CuT1 is characteristic [182]. In these cases, CuT1 is coordinated with three ligands corresponding to two histidine residues and one cysteine residue [183,184]. The triple coordination is presumably favoured because tetra-coordination reduces cationic exposure to the solvent, decreasing its polarization [185]. The absence of the fourth ligand is compensated by a strong Cu-S link, influencing the *E*_0_.

**Figure 2:**
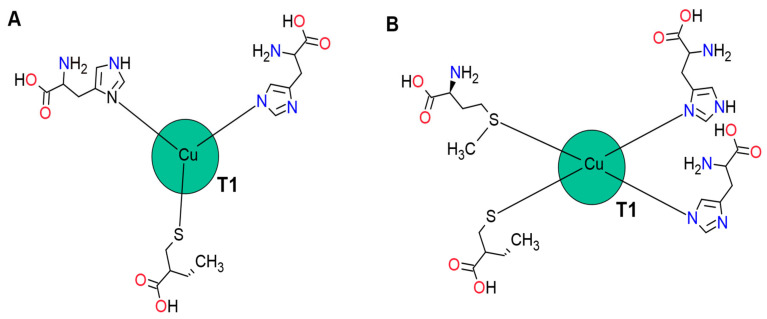
Coordination of ligand in T1 copper. (**A**) Coordination of copper and fungal laccases (tri-coordination). (**B**) Tetra-coordination in non-fungal laccases.

In laccases of bacterial origin, CuT1 may be tetra-coordinated to an axial ligand such as methionine or glutamine; in contrast, fungal laccases possess a non-coordinated residue such as leucine or phenylalanine instead of the axial methionine. These differences in coordination geometry in laccases influence the electronic structure and the transfer of electrons to CuT1 (Figure 2), [186]. 

CuT2 is a mononuclear copper site [20] that does not exhibit characteristic absorbance but has a hyperfine parallel coupling constant, similar to that of a typical tetragonal copper core [183]. This copper coordinated by two histidine residues and an oxygen atom as a hydroxide (OH^-^) ligand and is strategically located near the T3 copper, forming a coplanar trigonal configuration [176]. 

The CuT3 copper site is binuclear and consists of a pair of copper atoms identified as CuT3α and CuT3β [20,176]. CuT3 site have a weak UV absorbance (close to 330 nm) and no electronic paramagnetic resonance (EPR) signal, due to the antiferromagnetic (AF) coupling, that results from a ligand forming a hydroxide bridge between the coppers [183]. Each of the copper on the site is coordinated to three histidine and shares the hydroxide group.

Although it is common for laccases to have all four copper atoms, some laccases that do not have the characteristic blue colour may have zinc or iron atoms instead of four copper atoms [167]. Yellow laccase is characterized by not generating the absorption spectrum produced by the CuT1 site [20]. For pure laccase of *Myrothecium verrucaria* NF-05 after a quantitative analysis, 3.08 ± 0.3 copper atoms and 0.95 ± 0.2 iron atoms per protein molecule were obtained; however, the research did not define the arrangement of the metal atoms and which copper was replaced by the iron atom [187]. For the pure laccase of *Phellinus ribis*, the UV-VIS spectrum did not show the typical CuT1 peak (near 600 nm). In quantitative analysis, were found 2.0 ± 0.3 zinc atoms and 0.9 ± 0.2 copper atoms per molecule. However, inductively coupled plasma mass spectrometry analysis showed that the enzyme contained zinc, copper and manganese ions [188]. Atomic absorption analysis of *Pleorotus ostreatus* pure laccase (POXA1), shows 0.7 ± 0.2 copper/mol, 0.7 ± 0.2 iron/mol and 0.2 ± 0.2 zinc/mol protein, which would suggest an isoenzyme with a copper/iron/zinc stoichiometry of 1:1:2 [189].

## 9. Catalytic Mechanism of Laccases

**Figure 3:**
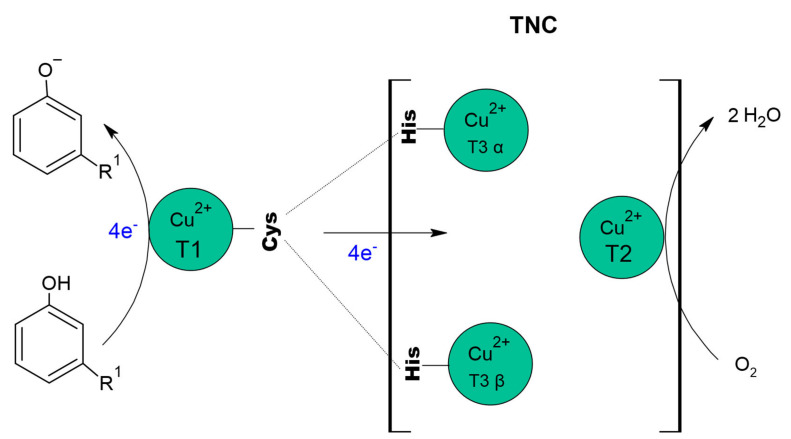
Simplified mechanism of the substrate oxidation reaction in laccases. Modified from [193].

It has been proposed that laccases must be resting with all copper in the oxidised state (Cu++), [179], with the four copper atoms representing the catalytic machinery of these enzymes [190]. The catalytic cycle in laccases begins with the oxidation of the substrate, CuT1 (Cu^++^) is responsible for sequestering electrons and transferring them to the trinuclear copper centre via His-Cys-His [20,178,179]. After oxidation of four electrons, is produced the fully reduced form (Cu^+^). Oxidation and intramolecular electron transfer are simultaneous to proton transfer, allowing the O_2_ reduction to H_2_O through ionizable groups within the TNC channels (Figure 3), [11,20,179,190,191,192]. 

The reaction mechanism of the laccases works like a battery, as it individually stores the electrons from the oxidation reaction to reduce molecular oxygen and produce water after receiving four electrons [11]. The use of oxygen in the enzymatic reaction has sparked interest in laccases at the industrial level; since O_2_ can be used as a primary oxidant, being possible to control the injection or decrease of O_2_ partial pressure during the enzymatic reaction can be controlled [194].

Besides the functionality of copper for the oxidation of different substrates, there is a critical structural parameter in laccases. The presence of a C-terminal glue in some laccases can act as a plug that blocks access to the TNC water channel, which has a variable extension of residues depending on the origin of the enzyme [191]. Giardina et al., (1999) performed an automatic sequence analysis of the pure POXA 1B C-terminal end, detecting its heterogeneity. During the study, they identified three sequences: -Leu-Pro-Ala-Pro-Leu-Lys (relative abundance, 40–45%); -Leu-Pro-Ala-Pro-Leu (40–45%) and -Leu-Pro-Ala-Pro (10–20%). The three C-terminal sequences generated from the same polypeptide chain and the heterogeneity observed could be explained by the existence of C-terminal processing of the protein or during the purification procedure [37]. Andberg et al., (2009) generates two mutants of *M. albomyces* MaL laccase, to evaluate the effect of the C-terminal end and found that the DSGL559 mutant (having a deletion of the last four C-terminal residues) affected the activity of the enzyme turning it almost inactive. In the mutation L559A that prevented C-terminal cleavage, changes in the geometry of the trinuclear site occurred [195]. 

## 10. Redox Potential of Laccases

Important laccases for industrial applications are those with high redox potential [196]. In laccases, the catalytic behaviour on most reducing substrates depends on the *E_0_* in CuT1, as this is the electron acceptor [197]. *E*_0_ is characteristic of each enzyme, ranging from ~0.400 to 0.800 V [198]. Laccases with low redox potential range from 0.340 to 0.490 V and commonly have methionine as an axial CuT1 ligand. Laccases with medium redox potential range from 0.470 to 0.710 V and have a non-coordinating leucine, and laccases with high redox potential range from 0.720 to 0.780 V and most have a non-coordinating phenylalanine [186].

On the other hand, laccases with high E0 have an ample distance of coordination between CuT1 and a His (Figure 2A) [182]. Increases in the coordination distance was observed in the structure of the *Trametes versicolor* TvL laccase caused by the formation of a hydrogen bond between two highly conserved residues (Glu460—Ser113), that generates the displacement of the helical segment (where is located the His458 residue), moving the N of the His458 residue away from the CuT1 [180]. A great distance between Cu-N (His) makes the Cu ion more electron-deficient due to a low N contribution, which destabilizes higher oxidation states, thus increasing the *E*_0_ [182,199]. 

Overall, the variations in the redox potential of CuT1 observed among laccases cannot be attributed to a single structural characteristic; it is the sum of factors such as the CuT1 coordination geometry and the nature of the waste that influence the accessibility of the solvent, the hydrogen bonds and the dielectric anisotropy [199].

The redox potential is a crucial parameter for enzymes, as the greater be the redox potential, the greater the range of substrates that can be transformed [200]. Therefore, the E_0_ restricts the substrates that the enzymes can oxidize. However, the oxidative capacity can be improved if adding mediators to the enzyme reaction, which could be an obstacle at the industrial level due to high cost and toxicity of mediators. For this reason, laccases with high redox potential are more attractive for biotechnological and environmental purposes [198]. 

## 11. Stability of Laccases

Stability studies use different analytical methodologies to identify the response of the enzyme exposed to various conditions. The most common analytical strategy for protein stability study is to determine kinetic and thermodynamic stability [201,202,203,204,205]. These studies help to identify the effect of different factors (temperature, pH, pressure and reaction inhibitors) on the activity and enzyme structure, generating results that facilitate the detection of the optimal conditions for the use and storage of the studied enzyme. Thermodynamic stability based on the free stabilization energy of the enzymes (∆G_stab_), where the difference between the free energies of the folded and unfolded states of the protein is support by the melting temperature of the protein (Tm); the temperature at which 50% of the protein unfolds. Kinetic stability depends on the energy barrier for irreversible inactivation and expressed as the half-life of the enzyme (t_1/2_) at a defined temperature. Both stabilities (kinetic and thermodynamic) correlate to the extent that they increase the resistance to splitting (higher Tm) and also increase resistance of the enzyme to inactivation (higher t_1/2_) [205,206]. 

Evaluating enzyme stability at different temperatures and pH has been one of the most frequent objectives. Li et al., (2016) study the effect of pH and temperature on the LS-10C laccase of *Trametes* sp., for which they incubated the enzyme at pH between 2.5–7.0 and between 20–70 °C for one hour, obtaining an optimum pH range of 4.0–4.5 and 40 °C as the optimum temperature [207]. Bertrand et al., (2016) found that the Lac6c laccase of *Trametes versicolor* after 120 min of exposure to temperatures between 25 °C and 70 °C showed variations in relative activity between 20% and 75%; however, they considered it very stable, as it maintained an average of 50% relative activity. Concerning to the pH they detected that it varied considerably, preserving 3%, 7%, and 28% of its relative activity at pH 3, 4 and 5, respectively, after 4 h of exposure [208]. For *Ganoderma lucidum* rGlCC1 expressed in *P. pastoris*, thermodynamic stability analyses performed during 1 h of exposure at different temperatures it was found that preserve its relative activity between 75 and 100%, in pH and temperature ranges from 2 °C to 11 °C and 10 °C to 60 °C, respectively [209]. Bao et al., (2013) found that laccase lac1 from *Coprinus comatus* maintained a residual activity of 51% after one hour at 60 °C. In terms of best pH for enzyme reaction, it varied depending on the substrate; resulting in pH of 3.0, 6.0, 5.0 and 6.0 for ABTS (2,2’azino bis (3-ethylbenzothiazolin-6-sulfonate)), guaiacol, DMP (2,6 dimethoxyphenol), and syringaldazine (SZ), respectively [210]. Authors demonstrated the importance of studying the stability of the enzymes, to establish the use and storage best appropriate conditions, so as the substrates on which the enzyme can act, as each isoenzyme has affinities that vary according to the conditions.

Several stability studies oriented to determine the conformational changes in a molecule under the influence of different factors under storage, known as the stability indicator method (SIM—acronym in English). This method evaluates the changes of the molecules without the interference of excipients, impurities and products that induce degradation, applied mainly to products of pharmaceutical use [211]. In general, when the stability of proteins and or enzymes is studied, it is usually done in three ways: in real-time [212], in an accelerated manner [213]], or computationally (computational dynamic simulation), [214,215]. In all cases, the study leads to the periodic monitoring of changes in the characteristics of the protein and or enzyme (biochemical, conformational, enzymatic activity, among others) over a long period, under the influence of different temperatures.

If the stability study is real-time [212], used temperatures do not significantly alter the characteristics of the enzyme; including the temperature at which the enzyme exhibits high enzymatic activity and maintains its native conformation. When the study is an accelerated stability study [213], conditions that cause stress to the enzyme, such as high temperatures (e.g., ≥40 °C), are often used. The dynamic computational simulation evaluates different temperatures that destabilise the enzyme and that presumably suitable for enzyme operation. Data processing identifies critical residues and regions for enzyme stability [214,215] and allows comparison with different laccases to determine which are significantly more stable [216].

A strategy to maintain or increase the stability and reuse of the enzyme is immobilization. For this purpose, supports are used that increase stability to adverse conditions such as pH, extreme temperature or the presence of organic solvents [217]. Laccases immobilization could be made by adsorption, mesh embedding, microencapsulated embedding, covalent binding, self-immobilization, and combinations of some of them [218]. 

The laccase immobilization by physical adsorption requires porous materials such as kaolinite, diatomite, activated carbon, montmorillonite, molecular sieve, porous glass, bentonite, silica gel, cellulose, graphene, carbon nanotubes, nanoporous carbon or fullerenes; while for ion adsorption requires materials such as agarose, chitosan or ion exchange resin [218]. Some examples of this type of immobilization employ some supports such as porous Purolite^®^ carriers [217] or multi-walled carbon nanotubes [219]; both methods showed increased storage stability, but differences in terms of thermo-stability [217,219]. The results demonstrated the importance of identifying the requirements and application requirements for each enzyme. Immobilization using the adsorption method has the disadvantage of decreasing the enzyme activity compared to the free enzyme but retains up to 80% activity after several cycles [220].

Immobilization using embedding materials includes natural gels such as chitosan beads, alginate, gelatin, agarose gels, and synthetic gels such as polyacrylamide and polyvinyl alcohol. Immobilizing laccases on materials such as calcium alginate has been reported to increase thermal stability and storage stability [218]. However, in some cases, the degradation of contaminants decreases when using the immobilized enzyme. Le et al., 2021 used copper alginate beads for commercial laccase from *T. versicolor* (Sigma-Aldrich) and found that immobilization affected the degradation of the containment agent between 16.7% and 34.0%, compared to the free enzyme [221]. Similar results obtained by Lassouane et al., 2019 by immobilizing crude laccase from *Trametes pubescens* MB89 on sodium alginate beads [222]. Although immobilization of enzymes is frequent, other investigations immobilized the wild-type or recombinant laccase-producing organisms [27,156].

## 12. Production and Heterologous Expression of Fungal Laccases

The most used laccases in the industry come from fungi, which often have high yields and produce several isoforms. However, the catalytic efficiency of each isoform is different [162]. In addition, obtaining laccases from the native fungus has two limitations, the first is related to the negative effect on the properties of the medium because the morphological growth affects the rheology of the broth and the second that the mycelium of the fungi can wrap around the impeller and spread along the feeding or sampling line. Some species of fungi (Basidiomycetes) produce high amounts of laccase; however, production may not meet industrial demand due to difficulties such as long growing periods [223], require greater control of growth conditions and demand high availability of oxygen [224]. This problem was observed in the study by Postemsky et al., (2017) when evaluating the production of *Ganodema lucidum* laccase in 3.5 kg of sunflower husk obtain only 16 Ug^−1^ [225]. On the other side, Patel et al., (2009) evaluated the laccase production of the fungus *Pleurotus ostreatus* in 5 g of wheat straw with copper, finding that it reached 14,189 Ug^−1^ of laccase after eight days of incubation [226]. The difficulties in producing and obtaining enzymes have led to 90% of industrial enzymes obtained from genetically manipulated microorganisms (heterologous expression) [227]. 

Laccases have expressed in hosts as *Saccharomyces cerevisiae* [228], *Yarrowia lipolytica* [229], *Pichia pastoris* [44,204,230,231,232], *Kluyveromyces lactis* [233] and even filamentous fungi such as *Aspergillus niger* [234], *Aspergillus sojae* [235], and *Trichoderma reesei* [236]. Even so, *P. pastoris* is the most successful expression host used in the production of recombinant proteins, mainly for biopharmaceuticals and industrial enzymes [41,42] and one of the most used for the expression of fungal laccases (Table 6). The reasons that influence the use of *P. pastoris* are: its high level of expression, the ability to use different carbon sources [43,237], the possibility of generating high cell density cultures, the production of large quantities of recombinant proteins [162], the positive response to different fermentation strategies [43,44,238], the high extracellular expression of recombinant proteins [239], is a GRAS (general recognized as safe) microorganism [240], post-translational modifications including polypeptide folding, glycosylation, methylation, acylation, proteolytic cleavage and targeting of subcellular compartments, the ability to design secreted proteins that can be easily separated from biomass [241] and the absence of the enzyme α-1,3-mannosyl transferase (EC 2.4.1.132) which produces the mannosyl bonds α-1,3 characteristically of *S. cerevisiae* and which are not desirable, especially for the production of recombinant proteins used in the pharmaceutical industry due to the hyperglycosylation pattern it generates in the cloned protein [242]. 

The industrial use of laccases requires that production conditions be optimized. One of the strategies to increase recombinant laccases production is to use strong promoters in gene construction. In *P. pastoris*, the most widely used promoter is the alcohol oxidase promoter (*pAOX*) [45,162] that methanol induced; however, methanol is toxic, can affect enzyme stability, increase proteolytic activity and generate difficulties in enzyme folding [162,253]. *pGAP* (the constitutive promoter of glyceraldehyde-3-phosphate dehydrogenase, E.C. 1.2.1.12) has successfully used for fungal laccases production [44,162,204,232]. 

The expression of recombinant proteins in *P. pastoris* under *pGAP* eliminates the risk and diminish the cost associated with the storage and use of large volumes of methanol, significantly reduces heat generation, the need for oxygen during production [254], and allows the use of various carbon sources for expression [253,255].

## 13. Biotechnological Applications of Laccases

Laccase is considered the enzyme with the highest availability for different commercial applications [255]. Laccases catalyse chemical reactions on a wide range of substrates and their ability to oxidise substrates is related to the presence of phenolic groups as observed in the model substrate (lignin), [256]. In this regard, treatability studies have been carried out using microorganisms that produce laccases, recombinant microorganisms and laccases alone, even when combined with other chemical and physical treatments, such as sequential treatment with *T. versicolor* and TiO_2_ for the treatment of paper wastewater [257], the dye-removal (Reactive Black 5) analysis using *T. versicolor* immobilized on a swab [258], the evaluation of the adsorption and detoxification of triphenylmethane dyes (Crystal Violet and Malachite Green), using *G. lucidum* and *P. ostreatus* and the laccase concentrates rGlLCC1 (from *G. Lucidum* expressed in *P. pastoris*) and rPOXA 1B (from *P. ostreatus* expressed in *P. pastoris*) [203], the evaluation of microbial consortia (native fungi and bacteria, recombinant yeasts and concentrated laccase) in a pilot plant for the treatment of wastewater from biological staining [2,27], the evaluation of a secondary biological treatment of non-conventional textile wastewater with an active ligninolytic fungal biomass coupled to a tertiary physico-chemical TiO treatment or coupled to algae [27,259] and the evaluation of a fungal/bacterial consortium, to establish the potential for laboratory-scale removal under non-sterile conditions, using a synthetic liquid waste containing triphenylmethane and azo dyes [260].

However, phenolic compounds are typical substrates for laccases because their redox potentials (0.5 to 1.0 V) are low enough to allow electron extraction [256]. The catalytic mechanism of laccases on phenolic and inorganic compounds occurs by oxidation bonds cleavage, by oxidative coupling mechanisms [21] or by the monoelectronic oxidation of diphenols and aromatic amines, which occurs when an electron and a proton are removed from the hydroxyl or amino group; forming a phenoxy or amino radical [21,167].

Phenol is one of the most dangerous pollutants to the environment. It is crucial in the petrochemical industry, coking, plastics, paper, oil refineries, and phenolic resin. Treatment with laccases has investigated as a strategy for phenol removal [261]. Although the action of laccase is commonly related to phenolic compounds, the low specificity of laccases allows them to react with non-phenolic compounds such as dyes. For the above reasons, laccases are valuable in the delignification of lignocellulosic compounds, bio-pulping, bio-bleaching, the transformation of dyestuffs from the textile industry, dyestuff removal, wastewater treatment and degradation of different recalcitrant compounds [22,23,24,25,26,262]. Some other applications of laccases appear in Table 7. 

Despite the wide use of laccases, they are not always efficient in removing some compounds; this has prompted the employment of intermediates (mediators) as a degradation strategy [1]. Mediators favoured the active centre of the enzyme to interact with large substrates or substrates with a high redox potential [162]. Mediators are low molecular weight compounds that oxidation, generate cation radicals that oxidize more complex compounds through a redox cycle by mechanisms such as hydrogen atom transfer, electron transfer or ionic mechanisms [20,267]. Mediators can be of natural or synthetic origin. Natural ones can be obtained from fungal secretions (e.g., 3-hydroxy-anthranilic acid, HAA), from plant secondary metabolites (e.g., syringaldehyde) or obtained during microbial degradation of lignocellulose (e.g., vanillin). Synthetic mediators can be compounds included in the -N-OH group (e.g. HBT) or –N–O• (e.g., TEMPO) [19]. 

Activity of laccases increased with mediators such as ABTS (most widely used), 1-hydroxybenzotriazole (HBT), hydroxyphthalimide (HPI), 2,2,6,6-tetramethyl-1-piperidnyloxy (TEMPO), acetosyringone (ACS), violuric acid (VA), syringaldehyde (SA), and vanillin (VA) [15,267,276,277] (Table 7). Each mediator requires different optimal conditions for reaction with a target compound and can oxidize the substrate by some of the three reported mechanisms (hydrogen atom transfer, electron transfer and ionic mechanisms) [267].

Different studies have shown the effect of laccase on several contaminating compounds when using mediators [231,251]. Ashe et al., (2016) evaluated seven redox mediators following the three oxidative mechanisms: hydrogen atom transfer (SA, HBT, VA, VAN and HPI), electron transfer (ABTS) and ionic mechanisms (TEMPO) to improve the oxidation of traces of organic compounds using the impure enzyme of *P. ostreatus*; this work achieved a high degradation of phenolic compounds using the ABTS and VA mediators, while for non-phenolic compounds the VA and HBT mediators were more efficient [267]. The effectiveness of mediators probably depends on the chemical reactivity of the radicals formed after the initial oxidation stage. Furthermore, due to the instability of the inter-mediates (oxidized mediator) during the catalytic oxidation of non-phenolic compounds, they must be continuously replenished [19], which shows a limit to the effectiveness of mediators. However, in some studies, the use of mediators had the opposite effect to that required. Leme Ike et al., (2019) evaluated the degradation of anthracene using Lac1Lg, obtained from *Leucoagaricus gongylophorus*, finding that ABTS reduced 14% of the degradation; an effect attributed to secondary reactions that reduced the effectiveness of the treatment [263]. 

## 14. Degradation of Synthetic Dyes by Laccases

The removal of synthetic dyes is an application where laccases have demonstrated their potential [231,278]. Currently, are being produced worldwide, more than 10,000 different dyes and pigments (anthraquinones, azo [279,280], indigos [281], triphenylmethanes [203,282,283], and neolans [284]). These dyes are employed in food, paper, plastic, textile, cosmetics and biological industries for dyeing and printing processes [285]. 

The advantages of using laccases for dye removal are that they produce low amounts of sludge at low cost, as we remarked before [11]. However, during the treatment with laccases, many factors affect the discolouration percentage. Although, type of dye influence discolouration, it has been demonstrated that isoenzymes from the same organism generate different discolouration percentages. Zhuo et al., (2019) evaluated the discolouration rates of Malachite Green (MG), Remazol Bright Blue R (RBBR), Bromophenol Blue (BB) and Methyl Orange (MO) by using the *Pleurotus ostreatus* recombinant laccases LACC6, LACC9 and LACC10. Finding that the degradation efficiency and dye resistance varied considerably between each isoenzyme; the LACC6 laccase generated higher removal rates with 91.5%, 84.9%, 79.1% and 73.1% of discolouration for MG, RBBR, BB and MO, respectively. In contrast, laccases LACC10 and LACC9 achieved discolouration percentages between 71.1–54.8% and 7.1–67.9%, respectively, demonstrating that discolouration effectiveness depends on the isoform used [286]. Gu et al., (2014) evaluated the discolouration efficiency of *Coprinus comatus* laccases Lac3 and Lac4 on 13 dyes (Bright Blue Remazol R (RBBR), Bright Blue Reactive X-BR (BB X-BR), Bright Blue Reactive K-GR (BB K-GR), Bright Blue Reactive K-3R (BB K-3R), Orange Reactive 1 (RO), Reactive Red X-3B (RR X-3B), Congo Red (CR), Dark Blue Reactive KR (DBR KR), Coomassie G-250 (C), Malachite Green (MG), Bromophenol Blue (BB), Methyl Violet (MV), and Victoria Blue (VB)). They found that Lac3 had a higher discolouration efficiency than Lac4 except for BB dye, on which Lac4 obtained ~63% removal and Lac3 ~57%. Lac3 removed between ~ 67% and 90% of colour compared to the dyestuffs RBBR, BB K-GR, DBR KR and between ~33% and 48% compared to the dyestuffs BB X-BR, CR, C, VM and VB. Finally, the dyes that were at least degraded (~10% removal) were RO and RR X-3B, showing that differences between enzymes and between dye structures influence discolouration [245].

Fan et al., (2011) evaluated the discolouration of MO, MG, BB and Violet Crystal (CV) using the supernatant of *Trametes* sp. 48,424 and the recombinant enzyme rLAC48424-1 from *Trametes* sp. The study found that the discolouration of MG BB and CV was ~97, 90 and 68% removal for both enzymes, but the recombinant enzyme rLAC48424-1 increased the discolouration of MO by 14%; even so, the discolouration of CV was very low, and they indicated that the use of mediators (ABTS) as a strategy could increase the discolouration [246]. 

Colao et al., (2006), using the recombinant lcc1 laccase from *Trametes trogii* evaluated the discolouration of Alizarin Red (AR), Carmoisine (CM), Cochineal Red (CCR), Sunset Yellow (SY), Patent Blue (PB) and Indigo Blue (BI) by using or not the addition of the VA mediator. Authors found that the use of the mediator increased the discolouration of the dyes AR, CM, CCR, SY, PB and BI by ~23%, 87%, 75%, 67% and 81%, respectively [287]. However, mediators are not always as efficient, and discolouration differences with or without the addition of the mediator could determine their use. Gu et al., (2014) assessed GM discolouration in the presence and absence of the HBT mediator with discolouration of 89.7% and an increase in discolouration of only 6.6% when the mediator was applied [245]. The use of mediators implies an additional cost and is not always sustainable [288], can participate in self-reactions producing toxic products that inhibit the growth of some bacterial strains [74]; unless the increase in discolouration is significant once the cost and benefits have evaluated.

The concentration of dyes, pH and temperature are some of the other factors affecting degradation. Hadibarata et al., (2012), evaluating the discolouration of Brilliant Blue Remazol R (RBBR) by using *Polyporus* sp., S133 laccase, found that at ≤300 mg L^−1^ of dye it was discoloured up to 90%, while only 75% colour removal when the concentration was between 400 and 500 mg L^−1^. They also found that the optimum pH was 5.0 ± 0.2 and that higher or lower values resulted in poor discolouration [114]. Forootanfar et al., (2016), using the laccase of *Paraconiothyrium variabile*, evaluated the discolouration and the effect of pH and temperature on the discolouration of Acid Orange 67 (AO), Disperse Yellow 79 (DY-79), Basic Yellow 28 (BY), Basic Red 18 (BR), Direct Yellow 107 (DY-107) and Direct Black 166 (DB); obtaining a discolouration of 65.3%, 53.3%, 46.7%, 40.7%, 34.0% and 26.2%, respectively. When evaluating pH and temperature independently of the dye class, the optimum pH was 5.0 ± 0.2 affecting the discolouration percentage. When evaluating the temperature, they found that the optimum for the dyes DY-79 (60%), AO (71.3%) and BY (58.4%) was 45 °C and for the dyes BR (48%), DY-107 (50%) and for DB it was 55 °C [23]. Although temperature influences the percentage of discolouration, the feasibility of using higher temperatures during treatments could be a problem because maintaining constant temperatures different from room temperature could represent an additional cost for the industry.

For recognizing the factors that influence discolouration, it is necessary to understand the pathways through which the transformation of dyes occurs. Liquid chromatography coupled to mass spectrometry (LC-MS) has provided insight into the degradation mechanisms of dyes (such as azo, anthraquinone, triphenylmethane and indigo) as it was possible to identify the intermediate and transformation products obtained after different stages throughout the treatments [160,286]. 

**Figure 4:**
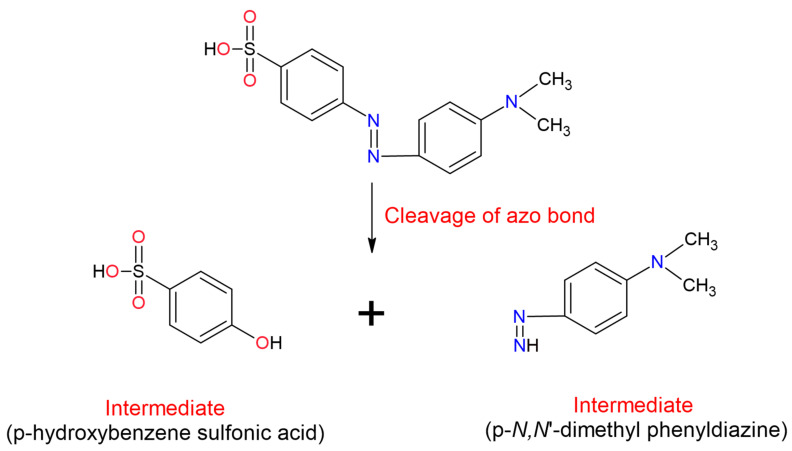
Route of degradation of azo dye Methyl Orange by laccase proposed by Telke et al., (2010). The excision of the dye is observed, and the immediate intermediate dyes are obtained [289].

The azo dyes degradation with laccases begins with the asymmetric excision of the azo bond followed by oxidative excision, desulfonation, deamination, demethylation or dihydroxylation, depending on the structure of the dye [160]. Discolouration of azo class dyes such as Methyl Orange (MO) has reported using *Aspergillus ochraceus* laccase NCIM-1146 [289], Congo Red (CO), with Tplac laccase from *Trametes pubescens* [280], Acid Orange 7 (AO7) using a laccase (not specified), [290]. In the three works, it was identified that in azo dyes, the first step in the discolouration is the splitting of the bond -N=N-, which results in obtaining two asymmetric intermediate products (Figure 4), [289,290,291]. The mechanism that the laccases carry out for the excision of azo dyes is through the formation of an electron-deficient reaction centre (carbocation). Carbocation generates highly reactive intermediates that are attacked nucleophilically (-OH, -SO or halogen ions) leading to an asymmetric cleavage of the azo bond [289].

**Figure 5:**
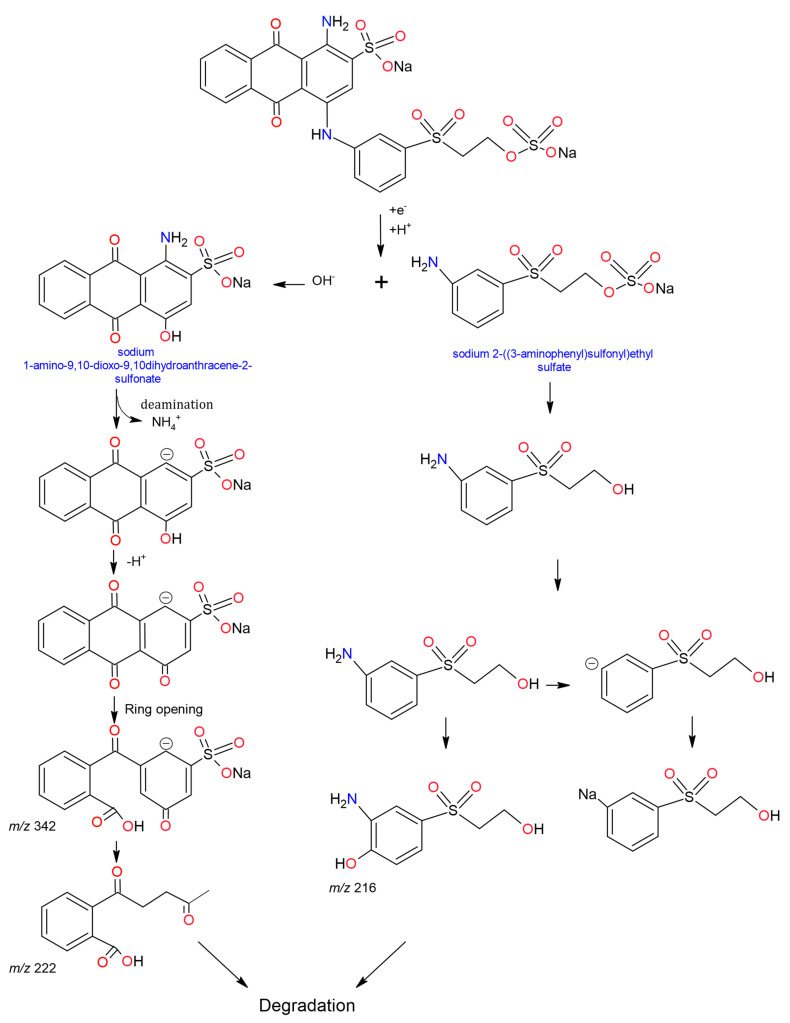
Route of degradation of the anthraquinone dye Remazol Brilliant Blue R (RBBR) by laccases. Adapted from [114,286,292].

Among the dyes of the anthraquinone class, perhaps one of the most evaluated has been RBBR. Osma et al., (2010) used the *Trametes pubescens* laccase to degrade RBBR and analyze the transformation products; and found the formation of two intermediaries after 2 h of treatment (*m*/*z* 304.30 and *m*/*z* 342.24), although no discolouration was yet observed, at the end of the treatment the formation of two by-products and the breaking of the chromophore was observed (Figure 5), [292]. Similar results obtained Hadibarata et al., (2012) when evaluating the transformation of RBBR with the immobilized laccase of *Polyporus* sp., found two intermediate by-products (*m*/*z* 304.3 and *m*/*z* 342.2) due to the break of the link -NH- that joins the chromophore of the dye. Furthermore, they indicated that the molecular weight of the metabolites was lower but that they are still toxic [114]. 

Navada et al., (2018), using the laccase of Phomopsis sp., found that the degradation of RBBR was generated by electron abstraction, forming an unstable intermediate radical, then occurs enzymatic oxidation, hydroxylation, deamination, to finally split the aromatic ring to obtain the intermediates *m*/*z* 229, 227, 336, 225 and 223 [293], but unlike Hadibarata et al., (2012), Navada et al., (2018), found that the low-mass compounds were not toxic. Zhuo et al., (2019), after treatment with *Pleurotus ostreatus* laccase HAUCC, proposed that the RBBR generates two by-products (*m*/*z* 324 and *m*/*z* 281) and the same processes mentioned were followed until the opening of the chromophore ring (Figure 5), [286]. Pype et al., (2019) analyzed it to compare methods of assessing RBBR discolouration using the commercial *Trametes versicolor* laccase. These authors identified that during degradation, the colour changed from blue to orange, a change attributed to the intermediate remaining with the undegraded anthraquinone chromophore (immediately after RBBR excision). In addition, they demonstrated an underestimation of the real degradation of up to 10%, caused by the methods of determining colour degradation [278]. 

For dyes of the triphenylmethane class have identified the metabolites and intermediaries formed during degradation with laccases. Yang et al., (2015) evaluated the degradation of MG mediated by lacA (*Cerrena* sp. HYB07). During degradation seven intermediaries resulting from the transformation were identified; among these, three were more persistent but decreased after prolonged incubation (tetradesmethyl MG (*m*/*z* 273.14), (methyl amino-phenyl)-phenyl-methanone (*m*/*z* 212.11) and (amino-phenyl)-phenyl-methanone (*m*/*z* 198.09)). Based on the results obtained, the model of MG degradation with two parallel pathways has proposed. Pathway I start with successive N-demethylation. However, the initial N-demethylation does not decolourise the MG, for this to occur, further degradation or polymerisation must happen and lead to chromophore destruction. In Pathway II, MG is hydroxylated to carbinol form, which is rapidly degraded between the central carbon and the N,N-dimethylamino phenyl ring. However, this pathway has reported only in the presence of a mediator [12]. Zhuo et al., (2019), using the *Pleurotus ostreatus* HAUCC laccase to degrade MG observed that the degradation reached 91.7%. Analysis of the transformation pathways showed that both degradation routes occurred. However, for degradation Pathway II, they propose that the natural mediators present (unspecified small molecules) in the extracellular fluid could be facilitators of colour degradation [286].

## 15. Degradation of Real Coloured Effluents Using Lacasases

The use of laccases for real effluents treatment has been little studied. The argument supposes that peroxidases and laccases are unsuitable for real-wastewater treatment due to their fast inactivation [294]. The removal of RBBR (artificially added) in real-wastewater was between 37.6% and 54.8% in 24 h. The variation was due to the different types of wastewaters used [221]. Ardila-Leal et al., (2020) performed colour removal in laboratory coloured wastewater using an enzyme concentrate of the recombinant enzyme (rPOXA 1B) from *Pleurotus ostreatus* and achieved 96% removal in 72 h [27]. The results demonstrate that laccase use is a potential strategy for the removal of laboratory dyes.

## 16. Conclusions 

The use of dyes marked the history of progress, evolution, cognitive and technological development and humanity. In prehistoric times, colours were extracted from natural sources (coal, ochre, plants or insects), causing a low pollution level. The development of civilizations favoured the spread of synthetic dyes, but later most of them were identified as highly toxic. The discovery of synthetic dyes changed dye production and ways of use, generating industrial development, but increasing environmental pollution levels. Synthetic dyes are poorly biodegradable, harmful to the environment, cause mutations, metabolic alterations, have carcinogenic effects and bioaccumulate. All of this affects organisms living in water bodies and human beings. For these reasons, wastewater treatment has implemented as a mechanism to solve the damages caused by humans, to reduce the pollutant load. Physical, chemical and biological treatments are strategies for colour removal. The most suitable removal of colour in wastewaters depends on the advantages of each type of treatment, the volume of wastewaters to treat, and the type and concentration of the dyes. However, enzymes (biological treatment) have gained interest due to their low impact on the environment. Among the enzymes chosen for this type of treatment, laccases can degrade different synthetic dyes, which allow for more environmentally friendly processes with less impact. Although laccases are enzymes with the potential for dyes removal and many other pollutants, studies are necessary to deepen their characteristics and properties to expand the technology. The evaluation of the redox potential, the use of mediators, the dye concentration, the dye structure and chemical class, the reaction pH and temperature, the stability of the enzyme, the reuse of the enzyme and the by-products generated in the enzymatic reactions are necessary to recognize and solve the difficulties in the diversification of the use of enzymes at industrial scale. In addition, some studies have shown that laccases can remove dyes in real wastewaters, which offers a complete picture of the use of laccases.

## Figures and Tables

**Table 1:** Classification and characteristics of dyes depending on the structure of the chromophore.

Dye Chemical Classes	Chromophore Structure	Examples of Dyes	Characteristics	Reference
Azo	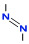	Methyl Orange	Azo dyes are frequently used (60%). These dyes have a functional group (-N=N-) linking two alkyl or aryl radicals, symmetrical and or asymmetrical, identical or non-azoic.	[87]
		Congo Red		
		Orange G		
		Amaranth		
Anthraquinone	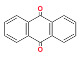	Remazol Brilliant Blue R	Anthraquinone dyes are the second most widely used dyes due to their low price, accessibility and performance in the dyeing process. They have anthraquinone chromophore groups comprising two carbonyl groups on either side of a benzene ring.	[88]
		Reactive Bright Blue X-BR		
		Reactive Blue 4		
		Alizarin Red S		
Triphenylmethane	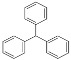	Malachite Green	These molecules have a central sp3 hybridised carbon atom, bonded to three aryl groups and belong to the most commonly used synthetic dyes in the textile industry.	[89]
		Crystal Violet		
		Bromophenol Blue		
		Light Green SF		
Nitro and Nitroso	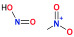	Naphthol Yellow S	In nitro dyes, a nitro group conjugates to an electron donor group via an aromatic system. Nitro dyes always contain a hydroxyl group as a donor.	[90]
		Disperse Yellow 26		
		Disperse Yellow 14		
Indigoid	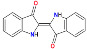	Indigo Carmine	Synthetic indigo is the most widely used dye in the textile industry worldwide. It is highly resistant to light and high temperatures.	[91]
		Ciba Blue 2B		
Xanthene	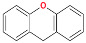	Rhodamine 6G	Xanthenes are dyes used in the food, cosmetics, paper and ink manufacturing industries because of their superior dyeing and colouring properties, but are poorly biodegradable, and some of them are very toxic.	[92]
		Rhodamine 123		
		Fluorescein		
Acridine	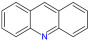	Acridine orange	Acridine dyes are heat-resistant, although they have low lightfastness. They are currently not very important commercially.	[93]
		Basic Yellow 9		
Phthalein	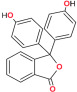	o-cresolphthalein	Phthalein dyes are employed to titrate weak acids. Phthalein dyes are insoluble in water but soluble in alcohol. There are frequently in the construction, coatings, electronics and electrical industries.	[94]
		Thymolphthalein		
		Dixylenolphthalein		
		Phenolphthalein		

The chemical structures were elaborated in the software ACD/ChemSketch, version 2020.1.2, (ACD/ChemSketch, version 2020.1.2, Advanced Chemistry Development, Inc., Toronto, ON, Canada, www.acdlabs.com, 2020 (accessed on 27 May 2021)).

**Table 2:** Some important aspects of the applications of synthetic dyes in the service sectors and in industry.

Sectors	Dyes Used	Applications	Characteristics	Referencias
Service providers (hospitals and universities)	Dyes used between the sector differ depending on the process of application.	Biological staining techniques, colouration of pharmaceuticals, staining of cells and for chemotherapy process as the detection of lymph nodes, and the location of the tumor lesions.	Volume of dye solutions is relatively small but with a concentration very high (~1 to 10 g L^−1^).	[78,95,106,107]
Textile	The textile industry uses a large variety of synthetic dyes. However, Azo dyes are the most employed. Further, textile industry dyes are classification according to their industrial application as acid, basic, reactive, vat, disperse and direct dyes.	Cellulosic fibers account for the highest world textile consumption. The different dyes applied depends on properties such as affinity for the fibre, diffusion, reactivity and stability of the bond between the dye and the fibre.	This industry is the greatest generators of colored effluents. The affinity of dyestuffs to cellulosic fibers relies on the nature of chromophore. Textiles industry consumes around 80% of the total production of dyestuff.	[4,9,87]
Printing	Dyes used vary according to the type of the fiber. For example, in polyester fabric printing, disperse dyes are the only dyes available.	Application of dyes to a restricted area on the fabric, paper, cardboard that is selected for applying the abstract of the design. Manufacture of decorative trims in paper.	Printing industry delivers a reduction of waste products this benefits for the printing facilities through reducing the number of raw materials and inadequate high costs for waste disposal. Printing machines have broad applications associated to fiber and dyes.	[9,97,108,109]
Cosmetics	The main components used in the cosmetic industry are pigments, although dyes are also used in small quantities. Over 80% of the colorants used are organic and more than 60% of them are azo-pigments.	Lipsticks, blushers, eye shadows, eyeliners and nail polish. Hair dyes, representing almost 80% of cosmetic products in Europe.	Methodologies for the determination of dyes in cosmetics are scarce with respect to those reported. The content of colorants in cosmetics vary widely depending on the type of product.	[78,110,111]
Food and Pharmaceutical	Currently, there are more than 60 known synthetic dyes for use in food. Some colorants (e.g., Tartrazine) must be used in accordance with the maximum allowable limit.	Carbonated drinks, fruit drinks, energy drinks, candies, cereals, desserts and snacks, among others.	Make products more attractive or to compensate for colour variations after food processing. Food and pharmaceutical industry dyes used are based on standards issued by the Codex Alimentarius Commission (CAC).	[99,100,104,105]
Leather and Tanning	Most leather dyed is using azo dyes or metal complex dyes. However, the structural of dye may be different from textile dyes.	Leather dyeing operations.	Approximately between 1% and 10% of pigments used in leather industries are lost as waste and its varying concentration is function of tanning and dyeing technology.	[112,113]

**Table 3:** Negative impacts of azo dyes, anthraquinones and triphenylmenthane on wildlife and or cell cultures.

Chemical Type of the Dye	Dye	Negative Impacts (Environmental or Organisms)	Reference
Azo	Direct blue 15 (DB15)	Larvae (*Danio rerio*) exposed to 100 to 500 mg L^−1^ showed that 96 h and 144 h after fertilisation developed a yolk sac oedema, curved tails and skeletal deformations. In addition, once degraded DB15, it produces carcinogenic amines.	[133]
		Azo dyes inhibit the proliferation of renal epithelial cells in vitro and, depending on the dose, cause a decrease in viable cells.	[134]
	Direct Black 38 (DB38)	The dye induced DNA damage at 250 mg L^−1^ after 48 h of exposure. This evaluation in a *Daphnia magna* model provided insight into the impact of the dye on the marine ecosystem.	[135]
	Disperse Yellow 7 (DY7)	Decreased survival of big-headed fish (*Pimephales promelas*) larval; some fishes succumbed between 4–10 days after hatching. The study employed 25.4 and 16.7 mg L^−1^ of DY7 and SRG respectively.	[125]
	Sudan Red G (SRG)		
	Disperse Red 1 (DR1)	The Ames test with DR1 and DR13 showed mutagenic activity for all *Daphnia similis* and *Vibrio fischeri* strains tested. Both dyes DR1 and DR13 were classified as very toxic to aquatic life.	[121]
	Disperse Red 1 (DR13)		
Anthraquinone	Erythrostominone (Ery)	Exposure to Ery caused a significant reduction in the quantity of *Daphnia magna* neonates produced from the second to fifth brood.	[136]
	Reactive blue 4 (RB4)	Reagent Blue 4, reduced (30–40%) *Triticum* sp. root length after exposure to 50–200 mg L^−1^. Was quantified with a 46.7 and 55.0% reduction in cell viability of HaCat and FHM cell lines, respectively. The dye was considered phytotoxic, cytotoxic and genotoxic.	[128]
	Vat Green 3 (VG3)	VG3 had an EC50 of 6.9 mg L^−1^ in *Daphnia similis* and an IC50 of 0.5 mg L^−1^ in *Pseudokirchneriella subcapitata*. Additionally, after 30 min of wastewater treatment with Photo-Fenton, the products generated were much more toxic than the original dye.	[137]
	Disperse Blue 3 (DB3)	Exposure of protozoa (*Tetrahymena pyriformis*) to dye DB3 increased by four hours the mean generation time, resulting in a reduction of bead ingestion by 70%. The Ames test showed a toxic effect only after metabolic activation of the protozoan.	[126]
Triphenylmethane	Malachite green (MG)	Exposure of *Hemichromis bimaculatus* (jewfish) to 13.5 mg L^−1^ MG caused mortality within 24 h, and exposure to a sublethal concentration (0.75 mg L^−1^) resulted in the loss of the escape reflex, sluggish lateral fins, increased irritability, wriggling, surface breathing, rolling and others.	[138]
		After 24 h exposure to 40 ng mL^−1^ of MG, leuco-malachite green (LMG) accumulation (~12.8% and 11%) was observed in the Zebrafish intestine and the ovary, respectively. Leuco-malachite green is a compound with carcinogenic, teratogenic and mutagenic potential.	[139]
	Crystal violet (CV)	Exposure of 40 ng mL^−1^ of CV for 24 h accumulated 14.5% of leuco-crystal violet (LCV) in the Zebrafish intestine. LCV is a cationic with a carcinogenic, teratogenic, and mutagenic potential.	[139]
		Assessments of eel muscle tissue *Anguilla anguilla* obtained from 91 sites in Belgian rivers showed a Crystal Violet detection frequency of 58.2%; this report shows the prevalence of dyes in wildlife.	[130]

**Table 4:** Some physical treatments used in the removal of dyes.

Treatment	Dye	Colour Removal	Parameters Influencing Treatment	Advantages	Disadvantages	References
Adsorption (activated carbon—adsorbent materials)	Light Green SF.	Associated with operating parameters.	Contact time, absorbent particle size, absorbent concentration, pH.	Between 75% and 86% of adsorption took place in the first 2 h.	Desorption processes are required to remove the dye, relatively high operating costs.	[129]
	Violet Cristal.	Between 72.2% and 97.8%. *	pH, adsorbent material, adsorption temperature, adsorbent surface area.	High adsorption capacity.	High temperatures (70 °C) are required, which increases operating costs.	[146]
Nanofiltration (composite membrane)	Congo Red, Methyl Blue, Sunset Yellow and Neutral Red.	Between 80.6% and 99.8%. *	Membrane composition and molecular weight cut-off, feed flow and dye loading.	High efficiency and possible dye reuse.	Membrane dye adsorption, high cost and required membrane cleaning procedures.	[147]
Flotation	Red 3BS, Navy SG, and Yellow S3R	Not specified	Agitation, type of gas sparger, range of pore	Efficient separation method for the removal of oil, dissolved ions, grease, biomolecules and solids suspended in water.	Pretreatments with coagulants or biosurfactants are required for dye removal.	[148]
Irradiation	Methylene Blue, Reactive Red KE-3B, Reactive Orange XBR.	Between 31.0 and 85.0%. *	Composition, hydrophilicity and porosity of the membrane, pH and salts, structure of the chromophore, irradiation time.	Degradation under visible light irradiation.	Use toxic solvents. The presence of salts decreases colour removal.	[149]

* Depends on the type of treatment and the type of dye.

**Table 5:** Some chemical treatments used in the removal of dyes.

Treatment	Dye	Colour Removal	Parameters Influencing Treatment	Advantages	Disadvantages	References
Ozonization	Coloured wastewater	25.0%	pH, ozone production, dye concentration.	Easy industrial application (on-site treatment) and no sludge generated.	High cost of energy consumption, low removal efficiency. Effluent quality.	[150]
Sonolysis	Coloured wastewater	15.0%	pH, sound power.	There is no additional sludge production.	High cost and energy consumption, low removal efficiency requires high volumes of dissolved oxygen.	[150,151]
Coagulation—flocculation	Acid Black 210.	93.2%	pH and the dose of the coagulant.	Simple and economical.	Some chemicals are toxic, sludge generation.	[149]
Electrochemical	Reactive Violet 5	Between 26.0% and 85.0% *	Initial dye concentration, current density, pH and electrolysisTime.	The decolorization achieved is rapid (only 70 min).	Efficiency depends on factors affecting removal efficiency.	[152]
Electrochemical oxidation	Synthetic effluent with 16 dyes	Between 13.9% and 94.0% *	Salinity, pH, type and concentration of electrolyte and treatment time.	Effluent discolouration is fast and efficient, easy to implement.	Process with high energy consumption, requires catalytic compounds.	[144]
Advanced oxidation process (UV/H_2_O_2_)	Reactive Green 19	99%	Irradiation level, pH, dye/H_2_O_2_ ratio, dye structure.	Easy handling, high stability, availability of H_2_O_2_, no sludge formation and high rate of mineralization.	Costly and undesirable products are generated.	[153]
Fenton and Fotofenton	Reactive Orange 4	Between 56.2% and 98.1%. *	pH, temperature, concentration of reagents	No complicated pressurized systems required for the oxidation process and the reagents are economical.	Requires acid pH (3.0), cannot remove disperse dyes and vat, high iron sludge generation.	[145,151,154]

* Depends on the type of treatment and/or the type of dye.

**Table 6:** Some fungal laccases heterologous expressed in *P. pastoris*.

Laccase	Origin of the Enzyme	References
rPOXA 1B	*Pleurotus ostreatus*	[44,204,230,232]
rGlLCC1	*Ganoderma lucidum*	[209,230]
lcc3	*Tremetes trogii*	[231]
ClLAC1I	*Colletotrichum lagenarium*	[243]
rLAC-EN3-1	*Ganoderma* sp.	[244]
Lac3/Lac4	*Coprinus comatus*	[245]
Lac1	*Cerrena sp.* HYB07	[239]
Lac1	*Coprinus comatus*	[210]
lac48424-1	*Trametes* sp. 48424	[246]
rLAC5930	*Trametes* sp. 5930	[247]
Lac2	*Lenzites gibbosa*	[248]
Lcc1A/Lcc1B	*Lentinula edodes*	[249]
lcc	*Pycnoporus sanguineus*	[250]
lacD	*Fome lignosus*	[251]
rLacD	*Trametes* sp.	[252]

**Table 7:** Some applications of laccases.

Origin of the Laccase	Application	Use of a Mediator	References
*Pleurotus ostreatus*	Obtaining bio-colourants	-	[217]
*Leucoagaricus gongylophorus*	Biodegradation of anthracene	ABTS	[263]
*Phanerochaete chrysosporium*	Degradation of polyhydroxyalkanoates into biosolids	-	[264]
*Moniliophthora roreri*	Hormone and anti-inflammatory degradation	-	[265]
*Trametes versicolor (Sigma)*	Biosensors to detect catechol	-	[266]
*Pleurotus ostreatus*	Degradation of traces of organic contaminants	HBT—HPI—SA—TEMPO—VA—ABTS—VAN	[267]
*Lentinus edodes, Pleurotus ostreatus, Ganoderma lucidum*	Biosynthesis of gold nanoparticles	-	[268]
*Cerrena unicolour*	Antibiotic degradation	ABTS	[269]
*Trametes versicolor (Sigma)*	Biocatalyst for enzymatic biofuel cells (EBC)	-	[270]
*Lenzites betulinus*	Degradation of organophosphates (chlorpyrifos)	-	[271]
*Trametes versicolor*	Isoproturon herbicide degradation	HBT—ACS	[25]
*Trametes versicolor*	Biosensors for quantification of pesticides in fruits	-	[272]
*Trametes versicolor (TvL)*	Biosensors for phenolic compounds	-	[273]
*Trametes versicolor* (commercial)	Organic transformations	-	[274]
*Aspergillus oryzae*	Biosensors	-	[275]

## Data Availability

Data is contained within the article.

## Abbreviations

AB80	Acid Blue 80
AB129	Acid Blue 129
ABTS	2,2′-azino-bis(3-ethylbenzothiazoline-6-sulfonic acid) diammonium salt
ACS	Acetosyringone
AO7	Acid Orange 7
AR	Alizarin Red
BB	Bromophenol Blue
BG	Bright Green
BB K-3R	Brilliant Blue Reactive K-3R
BB K-GR	Brilliant Blue Reactive K-GR
BB X-BR	Brilliant Blue Reactive X-BR
BI	Blue Indigo
BOD	Biochemical Oxygen Demand
CCR	Cochineal Red
CG-250	Coomassie G-250
CM	Carmoisine
COD	Chemical oxygen demand
CR	Congo Red
CV	Crystal Violet
DB3	Disperse Blue 3
DBR KR	Dark Blue Reactive KR
DMP	2,6 dimetoxifenol
DNA	Deoxyribonucleic acid
DWW	Domestic wastewater
*n*DWW	Non-domestic wastewater
FADH_2_	Flavín adenín dinucleótido (reduced form)
GRAS	General recognized as safe
HBT	1-hydrozybenzotriazole
HPI	N-hydroxyphthalimide
MG	Malachite Green
MV	Methyl Violet
NADH_2_	Nicotinamide adenine dinucleotide (reduced form)
NADPH_2_	Nicotinamide adenine dinucleotide phosphate (reduced form)
*pAOX*	Alcohol oxidase promoter
*pGAP*	Glyceraldehyde-3-phosphate dehydrogenase promoter
PB	Patented Blue
RBBR	Remazol Brilliant Blue R
RO	Reactive Orange
RO16	Reactive Orange 16
RR X-3B	Reactive Red X-3B
SA	Syringaldehyde
SZ	Syringaldazine
SY	Sunset Yellow
TDS	Total dissolved solids
TEMPO	2,2,6,6-tetramethyl-1-piperidinyloxy
TN	Total nitrogen
TNC	Trinuclear Copper Centre
TP	Total phosphorus
VA	Violuric acid
VAN	Vainillin
VB	Victoria Blue
